# Enhanced DNA and RNA pathogen detection via metagenomic sequencing in patients with pneumonia

**DOI:** 10.1186/s12967-022-03397-5

**Published:** 2022-05-04

**Authors:** Yukun He, Kechi Fang, Xing Shi, Donghong Yang, Lili Zhao, Wenyi Yu, Yali Zheng, Yu Xu, Xinqian Ma, Li Chen, Yu Xie, Yan Yu, Jing Wang, Zhancheng Gao

**Affiliations:** 1 Department of Respiratory and Critical Care Medicine, Peking University People’s Hospital, Beijing, 100044 China; 2 CAS Key Laboratory of Mental Health, Institute of Psychology, Chinese Academy of Sciences, 16 Lincui Road, Chaoyang District, Beijing, 100101 China; 3 Department of Psychology, University of Chinese Academy of Sciences, 19 A Yuquan Rd, Shijingshan District, Beijing, 100049 China; 4 Department of Respiratory, Critical Care, and Sleep Medicine, Xiang’an Hospital of Xiamen University, Xiamen, 361101 China

**Keywords:** Pneumonia, Metagenomic next-generation sequencing, Early pathogen detection

## Abstract

**Background:**

Metagenomic next-generation sequencing (mNGS) is an important supplement to conventional tests for pathogen detections of pneumonia. However, mNGS pipelines were limited by irregularities, high proportion of host nucleic acids, and lack of RNA virus detection. Thus, a regulated pipeline based on mNGS for DNA and RNA pathogen detection of pneumonia is essential.

**Methods:**

We performed a retrospective study of 151 patients with pneumonia. Three conventional tests, culture, loop-mediated isothermal amplification (LAMP) and viral quantitative real-time polymerase chain reaction (qPCR) were conducted according to clinical needs, and all samples were detected using our optimized pipeline based on the mNGS (DNA and RNA) method. The performances of mNGS and three other tests were compared. Human DNA depletion was achieved respectively by MolYsis kit and pre-treatment using saponin and Turbo DNase. Three RNA library preparation methods were used to compare the detection performance of RNA viruses.

**Results:**

An optimized mNGS workflow was built, which had only 1-working-day turnaround time. The proportion of host DNA in the pre-treated samples decreased from 99 to 90% and microbiome reads achieved an approximately 20-fold enrichment compared with those without host removal. Meanwhile, saponin and Turbo DNase pre-treatment exhibited an advantage for DNA virus detection compared with MolYsis. Besides, our in-house RNA library preparation procedure showed a more robust RNA virus detection ability. Combining three conventional methods, 76 (76/151, 50.3%) cases had no clear causative pathogen, but 24 probable pathogens were successfully detected in 31 (31/76 = 40.8%) unclear cases using mNGS. The agreement of the mNGS with the culture, LAMP, and viral qPCR was 60%, 82%, and 80%, respectively. Compared with all conventional tests, mNGS had a sensitivity of 70.4%, a specificity of 72.7%, and an overall agreement of 71.5%.

**Conclusions:**

A complete and effective mNGS workflow was built to provide timely DNA and RNA pathogen detection for pneumonia, which could effectively remove the host sequence, had a higher microbial detection rate and a broader spectrum of pathogens (especially for viruses and some pathogens that are difficult to culture). Despite the advantages, there are many challenges in the clinical application of mNGS, and the mNGS report should be interpreted with caution.

**Supplementary Information:**

The online version contains supplementary material available at 10.1186/s12967-022-03397-5.

## Introduction

Pneumonia is a common respiratory condition and remains a major health concern worldwide with substantial morbidity and mortality [1, 2]. Various pathogens can cause pneumonia, including bacteria, viruses, fungi, and parasites. In a clinical setting, uncertain pathogen diagnosis inhibits timely and effective therapy. Thus, a rapid and accurate etiologic diagnosis that can facilitate rational antimicrobial treatment is urgent [3]. A number of methods have been developed for the pathogenic diagnosis of pneumonia, such as pathogen culture and direct/indirect immunofluorescence assays. Various culture-independent nucleic acid amplification tests could aid in the diagnosis of pneumonia, including polymerase chain reaction (PCR) and loop-mediated isothermal amplification (LAMP) [4]. However, all existing methods have limitations owing to the complex microbial communities and emerging novel pathogens. No plausibly causative pathogen is identified in approximately 20–60% of patients with pneumonia [5, 6]. Another increasingly popular detection method, metagenomic next-generation sequencing (mNGS), has been available to aid pathogen detection from cerebrospinal fluid (CSF), bronchoalveolar lavage (BAL) fluid, plasma and others [7–13] and has unique strengths in detecting known and unknown microorganisms comprehensively [12, 14, 15]. However, for pneumonia, differentiation of true pathogens from respiratory tract colonization and environmental contamination [16] and the improvement of pathogen detection performance are currently the challenges of mNGS [15].

**Fig. 1:**
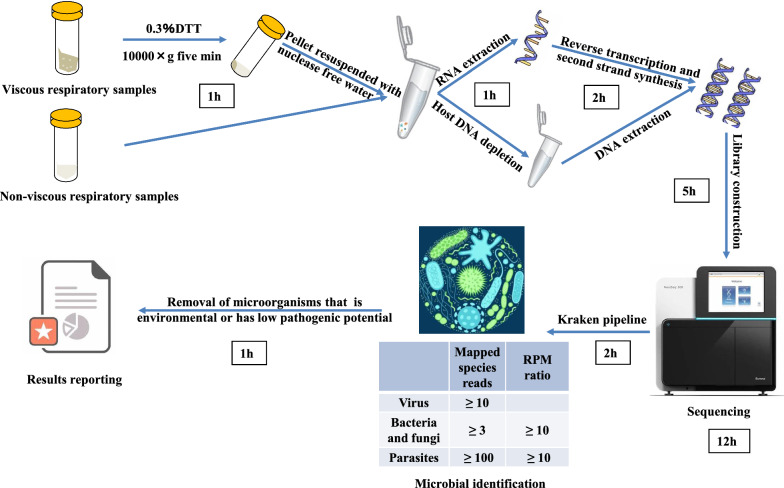
Complete mNGS assay workflow. A complete workflow for simultaneous DNA and RNA pathogen detection in different kinds of samples for pneumonia based on mNGS on one working day was developed. The pipeline includes sample processing, library preparation, sequencing, data processing, threshold criteria for pathogen detection and final results reporting

In this study, respiratory samples, including BAL or sputum collected from 151 patients with pneumonia, were detected using our pipeline based on the mNGS method. The pipeline consists of sample pre-treatment, nucleic acid extraction, library preparation, sequencing, and bioinformatics analysis (Fig. 1) and can simultaneously detect DNA and RNA pathogens within 1 workday. To improve detection performance, we optimized host DNA depletion and RNA library preparation procedures, and the pipeline was optimized by comparing the mNGS results with the control cases to remove background microorganisms. Moreover, we performed pathogen culture, LAMP assays, and viral qPCR according to clinical needs, aiming to evaluate the clinical utility and effect of our mNGS pipeline in pathogen detection.

## Study design and methods

### Sample collection, storage, and microbiological tests

The study was approved by the Ethical Review Committee of Peking University People's Hospital (ID: 2012-45, 2019PHB033-01). Written informed consents were obtained from all participators or their next of kin for incapacitated patients or unconscious subjects who were unable to give informed consents. The CAP cohort was registered at ClinicalTrials.gov (NCT03093220).

A retrospective analysis was conducted on 151 cases in eight tertiary hospitals in Beijing, Zhengzhou, and Fuzhou from 2013 to 2019. Demographic, laboratory findings, treatment, and patient outcomes were also investigated. The inclusion criteria were as follows: (1) patients who were diagnosed with pneumonia; (2) respiratory samples and detection process passed quality control for mNGS. The exclusion criteria were as follows: (1) patients who refused to undergo the mNGS examination and (2) patients without clinical data [17]. Pneumonia was diagnosed based on a composite reference standard based on the diagnostic criteria of community-acquired pneumonia (CAP) [18] and hospital-acquired pneumonia (HAP) [19]. Seventeen samples from cases without pneumonia, lung cancer or any other respiratory diseases passed quality control for mNGS and were then involved as a control group and used as a background set to identify “background flora” in respiratory samples (Additional file 1: Table S1). BAL and sputum of patients were collected for routine microbiological examination according to the clinical characteristics, including routine culture and/or virus qPCR and/or LAMP assays. qPCR detection can only detect some specific viruses that are clinically difficult to culture as a supplement, such as influenza A, influenza B, and respiratory syncytial virus. The LAMP assay was conducted using the respiratory pathogenic bacterial nucleic acid detection kit (CapitalBio Corporation, Chengdu, China) for 12 common pathogens (Additional file 1: Table S2) [20]. The remaining specimens were stored at − 80 °C for mNGS.

### Sample processing and library preparation

Viscous respiratory samples, such as sputum samples and some BAL samples, were liquefied with 0.3% dithiothreitol (dithiothreitol dry powder dissolved by phosphate-buffered saline to 0.3% (m/v)) for 10–30 min at 25 °C. Samples were then centrifuged at 10,000×*g* for 5 min, and the supernatant was removed. The remaining pellet was resuspended in nuclease-free water. BAL samples that were not viscous could go directly to the next step. Each sample was divided into DNA and RNA workflow. The RNA workflow mainly detects RNA viruses, whereas the DNA workflow can detect DNA viruses, bacteria, fungi, parasites, and other pathogens.

For the DNA procedure to remove human DNA, we tested various nonionic detergents to selectively lyse human cells before DNA extraction combined with Turbo DNase (with greater catalytic efficiency and tolerance to salt). A commercial human DNA depletion kit (MolYsis) was used for comparison with our in-house method. The procedure for human DNA depletion in our in-house is as follows. Saponin (Sigma–Aldrich, no. 47036, Shanghai, China) was added to the sample at a final concentration of 0.1%. Then, the sample was vortexed for 10 s and incubated for 5 min at 25 ℃, followed by the addition of 10 × Turbo DNase buffer (Thermo Fisher Scientific, USA) to a final concentration of 1× and 2 μl Turbo DNase (Thermo Fisher Scientific, USA). The sample was gently mixed and incubated at 37 °C for 30 min. Then, EDTA (5 mM final) was added and incubated at 75 °C for 10 min to inactivate the endonuclease before proceeding to standard extraction.

Total DNA was extracted with the Maxwell® RSC Viral Total Nucleic Acid Purification Kit (Promega, USA) after pre-treatment with lysozyme (Sangon Biotech, Shanghai, China) and lyticase (TIANGEN Biotech, Beijing, China) and fragmented into 200- to 300-bp fragments with a Bioruptor Pico. DNA libraries were constructed using the VAHTS Universal Plus DNA Library Prep Kit for Illumina (Vazyme, Nanjing, China).

The Maxwell® RSC RNA Tissue Kit (Promega, USA) was used for RNA extraction. Reverse transcription and second-strand synthesis were performed using the NEBNext® Ultra II RNA First-Strand Synthesis Module and Non-Directional RNA Second Strand Synthesis Module. In these steps, the incubation at 65 °C for 5 min was replaced by fragmentation at 94 °C. After DNA purification with Ampure XP beads, library preparation was performed using the TruePrep® DNA Library Prep Kit V2 for Illumina. Two other commercial RNA library preparation kits (NEB UltraII and Vazyme TR503) were used to compare the detection performance.

### Sequencing, data processing, and reporting of results

Libraries were sequenced to a depth of 5–15 million single-end, 75-base-pair reads on an Illumina NextSeq CN500 platform. The high-quality sequencing data were generated by removing low-quality reads, short reads (< 50 nucleotides) and low-complexity reads using fastp [21], followed by computational subtraction of human host sequences mapped to the human reference genome (hg19 and hg38) using BWA-short [22] alignment. After quality filtering, each nonhuman read was classified and assigned a taxonomic label by aligning to four microbial genome databases (bacteria, fungi, viruses, and parasites) that were downloaded from the National Center for Biotechnology Information (NCBI, version 20,200,723) (ftp://ftp.ncbi.nlm.nih.gov/genomes/) using Kraken 2 [23]. The database contains 5167 bacteria, 6268 viruses, 2022 fungi, and 341 parasites. The classified reads were processed for further data analysis.

Sterile deionised water was extracted with the specimens as a negative control (NTC). Detection of pathogens was reported automatically in an Excel sheet format based on pre-established threshold criteria [9, 24], including the reads per million (RPM) ratio (defined as RPM_sample_/RPM_NTC_) and mapped species reads (Additional file 1: Table S3). Following automatic pathogen detection, provisional reports were reviewed by a laboratory physician to verify the results. According to the results of the control group, which was used as a background set (Additional file 1: Table S4), a list of species was generated as “background flora” (Additional file 1: Figure S1), including *Veillonella*, *Rothia*, *Prevotella*, and *Fusobacterium*, which were reported to colonize respiratory microbiota [25, 26]. Microorganisms detected by mNGS that met the following criteria were identified as suspected pathogens: (1) pathogens of pulmonary infection, excluding the normal flora of the oropharynx or the skin (via a literature search against PubMed and the “background flora” list); and (2) meeting clinical judgment or targeted treatment response by two experienced clinicians.

Online Basic Local Alignment Search Tool (BLAST) alignment was used to examine the accuracy of the classified reads. The reads classified as certain organisms should have the highest confidence. Otherwise, the determined organism should be considered a false-positive result and would not be reported.

## Results

### Patient characteristics

In total, 151 patients were diagnosed with pneumonia, 97 of whom were males, with a median age of 55 years (36–68 years), 67 (44.4%) had underlying diseases, and 36 (23.8%) patients were diagnosed with severe pneumonia. Finally, 134 (88.7%) patients recovered and were discharged, and 10 (6.6%) patients died (Additional file 1: Table S4.1). The total leukocyte counts ranged from 2.0 to 5.4–109/L, while the percentages of lymphocytes and neutrophils were 0.2–64.9% and 26.6–97.6%, respectively. Normal values for total leukocytes, lymphocytes, and neutrophils were found in 75, 48, and 34 cases, respectively (Additional file 1: Table S4.2). The concentration of serum CRP was higher than 10.0 mg/L in 54 cases and higher than 0.5 μg/L in 16 cases for procalcitonin (PCT) (Additional file 1: Table S3.2).

### mNGS detection process optimization for respiratory specimens

Previous studies have shown that when applied to clinical practice, the diagnostic performance of mNGS in respiratory infection is unstable compared to different conventional tests [27, 28], which might be caused by the presence of commensal flora and variable pathogen features and loads [25, 29]. For respiratory specimens, mNGS detection has some limitations owing to the high proportion of host nucleic acids. By comparing different methods, we optimized the host DNA depletion procedure for our DNA workflow, and we found that saponin pre-treatment achieved a robust host DNA depletion effect when combined with Turbo DNase.

**Fig. 2:**
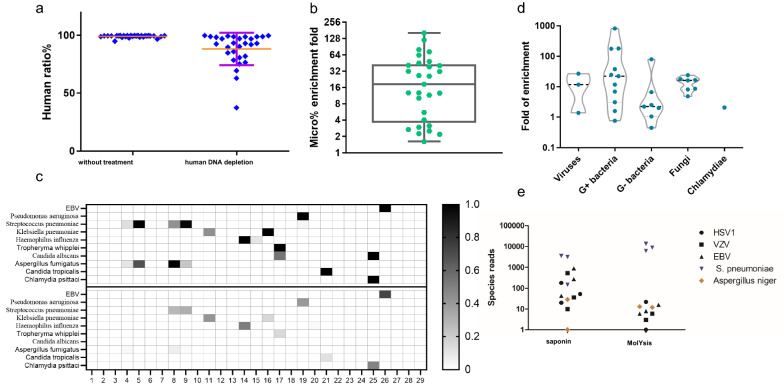
mNGS assay optimization on host DNA depletion. **a** The ratio of unique reads mapped to the human genome before and after human DNA depletion (mean with SD). **b** Relative enrichment of sequencing reads mapped to microorganisms by the host DNA depletion approach. **c** Pathogen detection in 29 samples without host DNA depletion (below) and after host DNA depletion (upper), shown by species RPM normalized by min–max normalization. **d** Relative enrichment of pathogen species reads before and after human DNA depletion in positive BALF specimens. Viruses (n = 3) are EBVs; G+ bacteria (n = 11) include *S. pneumoniae* and *Tropheryma whipplei*; G− bacteria (n = 7) include *Pseudomonas aeruginosa*, *Klebsiella pneumoniae* and *Haemophilus influenza*; fungi (n = 7) include *Aspergillus fumigatus*, *Candida albicans* and *Candida tropicalis*; and *Chlamydia* (n = 1) is *Chlamydia psittaci*. **e** Comparison of pathogen detection with two host DNA depletion methods. Three different BALF specimens spiked with HSV1, VZV, EBV, *S. pneumoniae* and *Aspergillus Niger* were undergo host DNA depletion with the saponin method and the MolYsis kit. After sequencing with 15 M for each library, species reads were calculated respectively

Twenty-nine BAL samples were subjected to mNGS analysis, both without host DNA depletion and with saponin depletion. For most samples, the original ratios of human reads were more than 99% and then reduced to approximately 90% (Fig. 2a). Reads belonging to the microbiome achieved an approximately 20-fold enrichment (Fig. 2b). Therefore, pathogens in BAL samples were more likely to be identified using the saponin depletion method (Fig. 2c). Without the host DNA depletion step, only 10 samples were positive. However, mNGS combined with the host DNA depletion step resulted in 19 positive samples. In addition, in three samples, only one pathogen was detected originally, but with the host DNA depletion step, more pathogens were detected.

Among these 19 positive samples, the enrichment efficiency of viruses, gram-positive (G+) bacteria, and fungi was observed with a tenfold increase (Fig. 2d), improving the detection capability of these pathogens. Notably, existing host depletion methods, such as MolYsis, achieved good performance on bacteria and fungi but sacrificed virus detection sensitivity. Our in-house host DNA depletion method exhibited an advantage for virus detection (Fig. 2e).

We also optimized RNA library preparation procedures to improve the RNA virus detection performance. Compared to the two commercial RNA library preparation kits, our in-house method showed a more robust RNA virus detection ability (Additional file 1: Figure S2).

### Comparison of mNGS and conventional tests in pathogen detection

#### The efficiency of mNGS in negative cases identified by conventional tests

At the time of clinical management, we performed pathogen culture for 103 patients, viral qPCR for 91 patients, LAMP assays for 111 patients, and then conducted mNGS for all 151 pneumonia patients (Additional file 1: Table S5). There were 72/103, 71/91, and 73/111 cases that failed to detect pathogens by culture, qPCR, and LAMP, respectively. In total, 76 cases failed to detect any pathogens, which indicates that unclear pneumonia would reach 50.3% under clinical management. However, among the 76 unclear cases, 24 probable pathogens were successfully detected in 31 (31/76 = 40.8%) cases using mNGS, which are all important pathogens of pneumonia, such as *Mycobacterium tuberculosis* complex (MTC), *Chlamydia psittaci*, and *Pneumocystis jirovecii*; however, these were difficult to identify through routine culture or directed PCR. Thus, our pipeline may greatly improve the detection efficiency in the negative cases identified by conventional tests (Additional file 1: Table S6).

#### Pathogen detection by mNGS relative to other methods

**Fig. 3:**
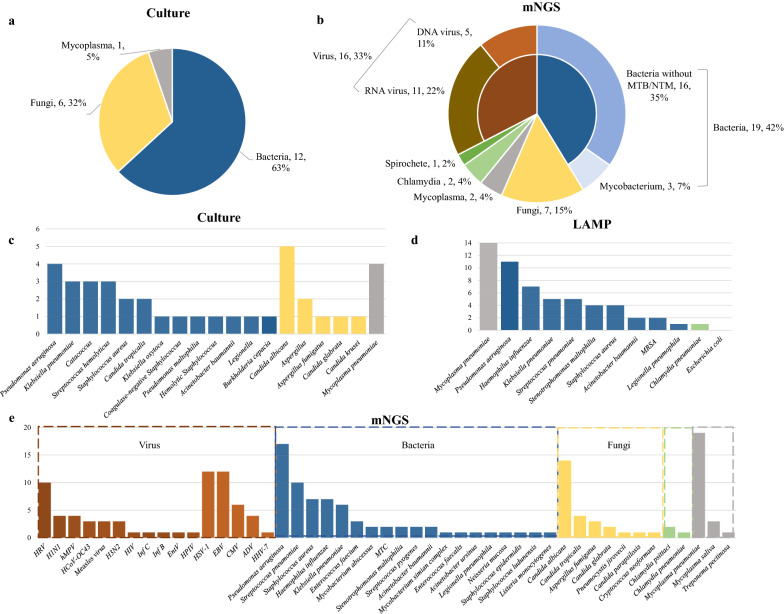
Comparison of culture, LAMP, and mNGS identification in terms of pathogen detection spectrum. Pathogen classification categories of culture and mNGS identification were displayed in **a**, **b**. Pathogen species and the corresponding number of cases identified by culture, LAMP and mNGS identification are shown in **c**–**e**, respectively. Different colours indicate different pathogen categories

In all 151 cases, 47 species were detected using mNGS (Fig. 3b), including bacteria, viruses, fungi, mycoplasma, chlamydia and spirochetes. In total, 19 pathogens were cultured (Fig. 3a), most of which were bacteria (12, 63.2%) and fungi (6, 31.6%). *Mycoplasma pneumoniae* was cultured in only four cases (Fig. 3c). Some common pathogens, such as *Mycobacterium*, could not be detected in routine culture. However, *Streptococcus hemolyticus* and *Burkholderia cepacia* were only detected through culture and not by mNGS. Viral PCR in the clinic depends on hypotheses and requires primers that may not always work and is limited to a very small portion of the genome [15]. Consequently, many types of viruses cannot be detected comprehensively by PCR. Thus, compared with conventional tests, mNGS has a broader spectrum of pathogens, especially viruses and other atypical pathogens. *Mycoplasma pneumoniae* tends to take a long time to be detected by culture and is the most commonly detected pathogen in mNGS and by LAMP, followed by *Pseudomonas aeruginosa* (Fig. 3d, e). However, *Candida albicans* was dominant in the culture detection (Fig. 3c).

The percentage of mNGS-positive patients was significantly higher than that of conventional testing-positive patients with regard to possible bacterial detection (*P* < 0.05), but no significant differences were found with regard to possible fungal detection (*P* = 0.492) (Additional file 1: Figure S3).

#### Concordance between mNGS and conventional tests

**Fig. 4:**
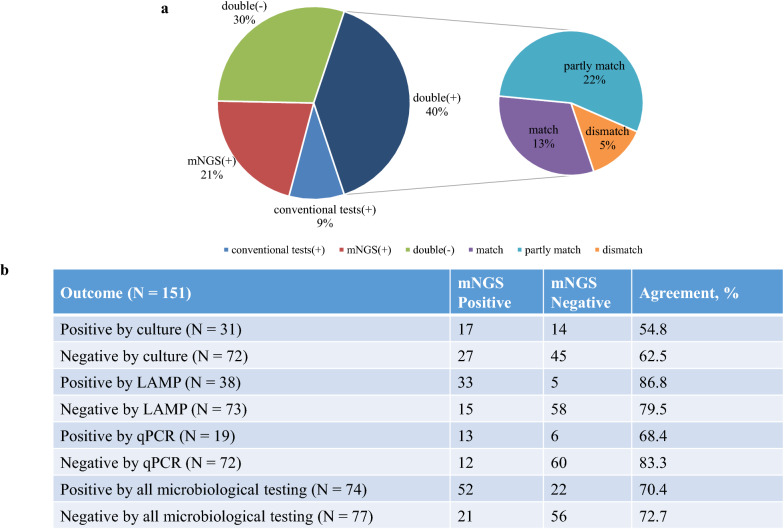
Concordance between metagenomic next-generation sequencing (mNGS) and conventional tests. **a** Pie chart demonstrating the positivity distribution for the detection of pathogens by mNGS and conventional testing in 151 cases. **b** Positive and negative agreement of mNGS versus culture, LAMP assay, qPCR and all conventional tests

In our results, mNGS and all conventional tests were both positive for pathogen detection in 60 (60/151 = 39.7%) cases and were both negative in 45 (45/151 = 29.8%) cases. A total of 32 (32/151 = 21.2%) cases were positive for pathogen detection by mNGS only, and 14 (14/151 = 9.3%) were positive by conventional tests only. Among the 60 double-positive cases, mNGS and conventional test results were matched in 19 cases and were totally mismatched in 8 cases. The remaining 33 cases were found to be partially matched, where at least one detected pathogen overlapped between mNGS and conventional tests (Fig. 4a). Taking all the microbiological test results as the gold standard, our in-house mNGS pipeline has a sensitivity of 70.4%, specificity of 72.7%, positive predictive value (PPV) of 71.2%, and negative predictive value (NPV) of 71.8%. The overall agreement was 71.5%.

We then compared the results of mNGS with those of the three conventional tests (Fig. 4b). The method for the comparison was based on a previous validation work [10]. Compared with the culture method, mNGS identified 55% of known pathogens in culture-positive cases, and it identified new probable pathogens in 47% of culture-negative cases. The agreement of the mNGS results with the culture results, LAMP results, and virus qPCR results was 60%, 82%, and 80%, respectively.

## Discussion

mNGS offers the possibility of fast pathogen identification without a prior hypothesis of the target [30] and has been employed for pathogen identification in various kinds of infection [11], mostly for CNS and respiratory system infections. According to the existing research, current clinical use of mNGS is more likely to be for “sterile” samples, such as CSF and blood [11, 31]. However, in respiratory samples (e.g., sputum, BAL), many attempts remain in the verification phase due to the complex microbial community. Meanwhile, the detection performance of mNGS in pneumonia is not completely consistent in multiple studies [17, 27, 32, 33]. Thus, although mNGS may have great potential for broad-spectrum surveillance or as a universal pathogen detection method, the results of mNGS should be interpreted with caution. In this retrospective study, we constructed a complete pipeline of microbiological tests based on mNGS and evaluated the detection efficiency of our pipeline with conventional tests in pneumonia.

Respiratory samples, especially BAL, have very low pathogen loads, and nearly all DNA content is host derived, thus limiting the overall analytical sensitivity of mNGS. We optimized the host DNA depletion procedure using saponin lysis, leading to the removal of host nucleic acids and the enrichment of reads belonging to the microbiome. Moreover, we optimized the RNA library preparation procedures, and the species reads were improved compared with two other commercial kits.

In our study, we systematically compared detection by mNGS and three conventional tests. First, mNGS was faster, taking an average of 24 h from processing samples to reporting, whereas the average feedback time of culture was at least three days [28]. Second, mNGS did not need a prior hypothesis and had a broader spectrum for pathogen detection than conventional tests (especially viral and rare infectious pathogens). Thus, mNGS might hasten clinical decision making and guide clinical laboratories to conduct targeted tests, which avoids the overuse of antibiotics for viral infections.

According to our data, mNGS may exhibit better performance than routine culture for detecting bacteria, whereas it was not superior to conventional tests for fungal detection (Additional file 1: Figure S4). Miao et al. reported that mNGS is not better than culture in recognizing bacteria but has superior feasibility in detecting fungi [28]. However, some studies reported inconsistent conclusions [17, 34, 35]. Possible explanations for this divergence are due to different sample types and some different test conditions of mNGS and culture.

Although approximately 12% higher than that of routine culture, the positivity rate (42.7%) of our pipeline seemed to be lower than expected. Previous studies reported a wide variety of sensitivities ranging from 40% [28, 31] to 97.2% [27] in pneumonia patients. The agreement of the mNGS results and culture results was 60%. In all 31 culture-positive samples, mNGS failed to detect pathogens in 14 samples, including fungi (6, 6/14 = 42.9%), G+ bacteria (5, 5/14 = 35.7%) and G− bacteria (3, 3/14 = 21.4%). Possible explanations may be due to difficult DNA extraction and some different test conditions of mNGS and culture, such as the step to break the walls for fungi and G+ bacteria. In addition, species reads of G− bacteria in some samples decreased after human DNA depletion compared with before (Fig. 2d), which may be the cause of false negatives. Meanwhile, due to the long-term storage and multiple freeze–thaw steps of some samples, some DNA may degrade. Through the comparison of mNGS and conventional tests in 11 samples collected within 6 months, the results indicated that mNGS had a higher sensitivity and a better performance (Additional file 1: Table S7). Thus, for mNGS, sample transport and storage are important, and the DNA extraction process still needs to be optimized for better enrichment of microbial DNA and increased detection efficiency.

With all three microbiological test results, the agreement was 71.5%. This may be associated with the limitations in our studies. Since this is a retrospective study, conventional tests for samples are limited and selected based on clinical characteristics. Some special important clinical cultivation tests and viral PCR, such as acid-fast culture for Mycobacterium tuberculosis, were not conducted. Thus, we suggest that mNGS could yield higher efficiency for the early detection of pathogens without a prior hypothesis of the target.

Other limitations of this study should also be noted. The potential pathogens detected by our pipeline did not mean that definite causing agents of the cases were found. The complete clinical information of the patients should be reviewed, and further validation should be conducted to identify newly identified pathogens. In our study, we involved two experimental clinicians to evaluate the detection of each species based on detailed clinical information. However, the clinical samples were so limited that validation tests could not be conducted in those samples with mNGS-only detection. Further research should include a larger sample size and prospective and controlled studies, which will help us better evaluate the clinical utility and value of our mNGS pipeline in the pathogen detection of pneumonia.

## Conclusions

mNGS is a revolutionary technology that harbours great potential for pneumonia but has limitations of high proportion of host sequences and irregular workflow. In this study, the hurdles were addressed and a complete, rapid, comprehensive mNGS pipeline was built to provide timely (approximately 1-working-day) DNA and RNA pathogen detection for pneumonia. The saponin and Turbo DNase pre-treatment is more conducive to microbial detection and our in-house RNA library preparation method had advantages in the detection of RNA viruses. Thus, our pipeline had a higher microbial detection rate and a broader spectrum of pathogens (especially for viruses and some pathogens that are difficult to culture). Despite the advantages, there are many challenges in the clinical application of mNGS, and the mNGS report should be interpreted with caution. Thus, similar to other clinical tests, the application of this new method in the clinic should be accompanied by rigorous clinical studies, and how, when to test this method requires further discussion.

## Supplementary Information

**Additional file 1.** **Supplement 1** The pretreatment of samples before DNA extraction in order to remove Human DNA;**Supplement 2** The bioinformatic analysis;**Supplement 3** Supplementary Tables;**Supplement 4** Supplementary Figures.

## Data Availability

The datasets presented in this study can be found in online repositories. The names of the repositories/repositories and accession number (s) can be found below: https://www.ncbi.nlm.nih.gov/, PRJNA741852. The data supporting the findings of this study are available by contacting the corresponding author.

## Abbreviations

BAL: Bronchoalveolar lavage
CAP: Community-acquired pneumonia
CSF: Cerebrospinal fluid
HAP: Hospital-acquired pneumonia
LAMP: Loop-mediated isothermal amplification
Mngs: Metagenomic next-generation sequencing
qPCR: quantitative real-time polymerase chain reaction
RPM: Reads per million
