# Supplementary material for: Enhanced DNA and RNA pathogen detection via metagenomic sequencing in patients with pneumonia
Source: J Transl Med. 2022 May 4;20:195. doi: 10.1186/s12967-022-03397-5 (PMC9066823; doi:10.1186/s12967-022-03397-5)
Supplement: Supplementary file 1 — Additional file 1. Supplement 1 The pretreatment of samples before DNA extraction in order to remove Human DNA;Supplement 2 The bioinformatic analysis;Supplement 3 Supplementary Tables;Supplement 4 Supplementary Figures. [file 12967_2022_3397_MOESM1_ESM.docx]

**Supplementary materials**

**Contents of Supplements**

[Supplement 1 The pretreatment of samples before DNA extraction in order to remove Human DNA 2](#_Toc88848891)

[Supplement 2 The bioinformatic analysis 2](#_Toc88848892)

[Supplement 3 Supplementary Tables 4](#_Toc88848893)

[Supplement 4 Supplementary Figures 44](#_Toc88848894)

# Supplement 1 The pretreatment of samples before DNA extraction in order to remove Human DNA

Saponin (Sigma, Shanghai, China) was added to the sample at 0.1% final concentration. Then the sample was vortexed for 10 s and incubated for 5 min at RT, followed by the addition of 10 × Turbo DNase buffer (Thermo Fisher Scientific, USA) to a final concentration of 1 × and of 2 μl of Turbo DNase (Thermo Fisher Scientific, USA). The sample was gently mixed and incubated at 37°C for 30 min. Then EDTA (5 mM final) was added and incubated at 75°C for 10 min to inactivate the endonuclease before proceeding to standard extraction.

# Supplement 2 The bioinformatic analysis

The high-quality sequencing data were generated by removing low-quality reads, short reads (< 50 nucleotide) and low-complexity reads using fastp, followed by computational subtraction of human host sequences mapped to the human reference genome (hg19 and hg38) using Burrows–Wheeler (BWA) alignment. The setting of the fastp was “--qualified_quality_phred 25 --length_required 50 --low_complexity_filter --unqualified_percent_limit 20”. The remaining data by removal of low-complexity reads were classified by simultaneously. After quality filter, each non-human read was classified and assigned a taxonomic label by aligning to four microbial genome databases (bacteria, fungi, viruses, and parasites) which were downloaded from the National Center for Biotechnology Information (NCBI version.20200723) (ftp://ftp.ncbi.nlm.nih.gov/genomes/) using Kraken 2. The database contains 5167 bacteria, 6268 viruses, 2022 fungi, and 341 parasites. The classified reads were processed for further data analysis.

# Supplement 3 Supplementary Tables

TableS1 The background set from 17 clinical samples including BALF and sputum

| Case ID | Sample ID | Sample Volume (μL) | Library Type | Raw Reads(M) | Raw Q30(%) | Clean Reads(M) | Uniq Reads(M) | Human reads-sum ratio(% by Uniq) | Classified Microbio reads(M) | Classified Microbio reads ratio(% by Uniq) |
| --- | --- | --- | --- | --- | --- | --- | --- | --- | --- | --- |
| 152 | 152 | 200 | DNA | 17.55 | 89.25 | 14.1 | 13.4 | 96.09 | 0.14 | 1.07 |
| 153 | 153 | 200 | DNA | 17.63 | 87.94 | 14.56 | 12.68 | 97.93 | 0.06 | 0.49 |
| 154 | 154 | 200 | DNA | 19.05 | 89.09 | 15.67 | 14.2 | 85.1 | 0.6 | 4.24 |
| 155 | 155 | 200 | DNA | 25.98 | 89.04 | 20.77 | 20.07 | 98.49 | 0.1 | 0.51 |
| 156 | 156 | 200 | DNA | 14.93 | 88.75 | 12.09 | 11.56 | 95.92 | 0.12 | 1.06 |
| 157 | 157 | 200 | DNA | 19.23 | 88.28 | 15.64 | 14.72 | 93.09 | 0.29 | 1.98 |
| 158 | 158 | 200 | DNA | 18.28 | 88.25 | 15.28 | 14.73 | 99.67 | 0.01 | 0.07 |
| 159 | 159 | 200 | DNA | 20.05 | 89.17 | 16.5 | 15.39 | 91.41 | 0.39 | 2.54 |
| 160 | 160 | 200 | DNA | 17.1 | 90.73 | 14.42 | 13.71 | 83.78 | 0.54 | 3.95 |
| 161 | 161 | 200 | DNA | 19.92 | 89.76 | 17.23 | 16.6 | 98.35 | 0.06 | 0.39 |
| 162 | 162 | 200 | DNA | 13.22 | 89.84 | 11.5 | 11.12 | 99.79 | 0 | 0.03 |
| 163 | 163 | 200 | DNA | 16.95 | 90.17 | 14.07 | 13.62 | 97.28 | 0.09 | 0.7 |
| 164 | 164 | 200 | DNA | 15.96 | 89.97 | 13.6 | 13.12 | 98.28 | 0.06 | 0.46 |
| 165 | 165 | 200 | DNA | 17.82 | 89.98 | 14.84 | 12.68 | 68.08 | 1.01 | 8.0 |
| 166 | 166 | 200 | DNA | 14.21 | 89.91 | 11.54 | 11.13 | 95.18 | 0.15 | 1.32 |
| 167 | 167 | 200 | DNA | 15.65 | 89.2 | 12.82 | 12.29 | 97.23 | 0.1 | 0.78 |
| 168 | 168 | 200 | DNA | 14.5 | 89.42 | 11.84 | 10.57 | 74.11 | 0.76 | 7.2 |

TableS2 The pathogens detected in the LAMP assay

| Pathegon |
| --- |
| *Acinetobacter baumannii* |
| *Chlamydia pneumoniae* |
| *Escherichia coli* |
| *Haemophilus influenzae* |
| *Klebsiella pneumoniae* |
| *Legionella pneumophila* |
| *Mycoplasma pneumoniae* |
| *Pseudomonas aeruginosa* |
| *Staphylococcus aureus* |
| *Stenotrophomonas maltophilia* |
| *Streptococcus pneumoniae* |
| *Methicillin-resistant Staphylococcus aureus* |

TableS3 The threshold criteria for different kinds of pathogens

| Pathegon | Mapped species reads | RPM ratio |
| --- | --- | --- |
| Virus | ≥ 10 | ≥ 10 |
| Bacteria | ≥ 3 | ≥ 10 |
| Fungi | ≥ 3 | ≥ 10 |
| *Mycobacterium tuberculosis* | ≥ 1 | ≥ 10 |
| Parasites | ≥ 100 | ≥ 10 |
| Other pathogens | ≥ 10 | ≥ 10 |

TableS4.1 Demographic and baseline characteristics of patients with pneumonia

| Patient characteristics | All patients (n = 151) |
| --- | --- |
| Age, years (median, IQR) | 55(36,68) |
| Male sex— no. (%) | 97(64.2) |
| Smoke— no. (%) | 32(21.2) |
| Underlying Illness — no. (%) | 67(44.4) |
| Diabetes | 14(9.3) |
| Hypertension | 22(14.6) |
| Cardiovascular disease | 13(8.6) |
| Chronic obstructive pulmonary disease | 1(0.7) |
| Bronchiectasis | 4(2.6) |
| Old pulmonary tuberculosis | 4(2.6) |
| Chronic liver disease | 7(4.6) |
| Renal disease | 6(4.0) |
| Cerebrovascular disease | 15(9.9) |
| Malignancy | 3(2.0) |
| Severe pneumonia— no. (%) | 36(23.8) |
| Ventilation— no. (%) |  |
| HFNC or noninvasive ventilation | 28(18.5) |
| Intubation and invasive ventilation | 11(7.3) |
| Outcome— no. (%) |  |
| Improved | 7(4.6) |
| Recovered and discharged | 134(88.7) |
| Death | 10(6.6) |

Abbreviations: HFNC, High-flow nasalcannulae

TableS4.2 Laboratory findings of patients with pneumonia

| Patient characteristics | Patients |
| --- | --- |
| White blood cell count, ×10^9^/L (n = 129) | 9.89 (2.0-25.4) |
| <4 | 14 (10.9) |
| 4-10 | 75 (58.1) |
| >10 | 40 (31.0) |
| Percentage of neutrophils (n = 103) | 76.6(26.3-97.6) |
| <40% | 1 (0.97) |
| 40–75% | 48 (46.6) |
| >75% | 54 (52.4) |
| Percentage of lymphocytes (n = 95) | 15.0 (0.7-64.9) |
| <20% | 57 (60.0) |
| 20–40% | 34 (35.8) |
| >40% | 4 (4.2) |
| CRP, mg/L (n = 68) | 92.5 (0.5-299) |
| <10 mg/L | 14 (20.6) |
| 10–50 mg/L | 18 (26.5) |
| 51–100 mg/L | 13 (19.1) |
| >100 mg/L | 23 (33.8) |
| Procalcitonin, ng/mL (n = 35) | 1.9(0.043-19.38) |
| <0.1 ng/mL | 7 (20) |
| 0.1–0.24 ng/mL | 5 (14.3) |
| 0.25–0.5 ng/mL | 7 (20) |
| >0.5 ng/mL | 16 (45.7) |

Data are presented as n (%) or means (range).

Abbreviations: CRP, C-reactive protein

TableS5 Pathogen detection using conventional tests

|  | Positive | Negative | Sum |
| --- | --- | --- | --- |
| Pathogen culture | 6 | 28 | 34 |
| qPCR | 0 | 1 | 1 |
| LAMP | 9 | 10 | 19 |
| Pathogen culture and qPCR | 2 | 3 | 5 |
| Pathogen culture and LAMP | 4 | 3 | 7 |
| qPCR and LAMP | 13 | 15 | 28 |
| All three conventional methods | 41 | 16 | 57 |
| Sum | 75 | 76 | 151 |

TableS6 Pathogen detection using mNGS and all conventional tests

| **Case ID** | **RNA virus** | **DNA virus** | ***Mycobacterium*** | **Spirochetes** | **Bacteria** | **Chlamydia** | **Fungi** | **Mycoplasma** | **Culture** | **PCR** | ***LAMP*** |
| --- | --- | --- | --- | --- | --- | --- | --- | --- | --- | --- | --- |
| 1 | HCoV-OC43(690) |  |  |  | *Pseudomonas aeruginosa* (922) |  |  |  | *Pseudomonas aeruginosa* | HCoV-OC43 | *Pseudomonas aeruginosa* |
| 2 |  |  |  |  |  |  |  |  | Aspergillus | HRV | *-* |
| 3 |  | HSV-1 (9) |  |  | *Pseudomonas aeruginosa*(534182) *Staphylococcus aureus*(220315) |  | Candida albicans(79) | *Mycoplasma saliva*(2381) |  |  | *MRSA Pseudomonas aeruginosa* |
| 4 | HRV(145) | EBV (267) |  |  | *Stenotrophomonas maltophilia*(1197) |  | Candida albicans(192) |  |  |  | *-* |
| 5 | HRV(10) | HSV-1 (402) EBV (35) |  |  | *Enterococcus faecium*(58514) *Staphylococcus aureus*(20672) |  | Candida albicans(1517) |  |  |  | *Staphylococcus aureus* |
| 6 |  |  |  |  |  |  |  |  | - |  |  |
| 7 |  |  |  |  |  |  |  |  |  | - | *-* |
| 8 |  |  |  |  |  |  | Candida tropicalis(13) Aspergillus fumigatus(6) |  | Candida albicans Candida tropicalis | INFA | *-* |
| 9 |  | ADV (4) | Mycobacterium abscessus (11) |  | Klebsiella pneumoniae（40） |  | Candida glabrata(1163) Aspergillus fumigatus(3) |  | *Klebsiella pneumoniae* | - | *Klebsiella pneumoniae* |
| 10 |  |  |  |  |  |  |  |  |  | - |  |
| 11 |  |  |  |  |  |  |  |  | - |  |  |
| 12 |  |  |  |  |  |  |  |  | - |  |  |
| 13 |  |  |  |  |  |  |  |  | - |  |  |
| 14 |  |  |  |  |  |  |  |  | - |  |  |
| 15 |  |  |  |  |  |  |  |  | - |  |  |
| 16 | HIV(63) | EBV (6897) HSV-1 (1779) CMV (312) |  |  |  |  | Candida albicans(21233) |  | - |  |  |
| 17 |  |  |  |  |  |  |  |  | - |  |  |
| 18 |  | ADV(26332) |  |  |  |  |  |  | - |  |  |
| 19 |  |  | Mycobacterium tuberculosis complex (MTC) (2) |  |  |  | Candida albicans(4) |  | - |  |  |
| 20 |  | HSV-1 (14553) EBV (18) |  |  | *Enterococcus faecalis*(134) |  | Candida albicans(120) |  | - |  |  |
| 21 |  |  |  |  |  |  |  | *Mycoplasma pneumoniae*(257) | - |  |  |
| 22 |  |  |  |  |  |  |  |  | - |  |  |
| 23 |  | CMV (4) |  |  | *Klebsiella pneumoniae*(153) |  |  |  | *Klebsiella pneumoniae* |  |  |
| 24 | HRV(350) |  |  |  |  |  |  |  | - | HRV | *-* |
| 25 | H1N1(35387) | HSV-1 (16) |  |  |  |  | Candida albicans(33) |  | - | INFA | *-* |
| 26 |  |  |  |  |  |  |  |  | - | - | *-* |
| 27 | H1N1(563) |  |  |  |  |  | Candida albicans(162) |  |  | INFA | *-* |
| 28 |  | CMV (422) |  |  |  |  | Pneumocystis jirovecii (108661) |  | - | - |  |
| 29 | hMPV(12514) |  |  |  |  |  |  |  | - | hMPV | *-* |
| 30 | Inf C(29) |  |  |  | *Klebsiella pneumoniae*(318) |  |  |  | Candida albicans | - | *-* |
| 31 |  |  |  |  | *Klebsiella pneumonia*e(71) *Acinetobacter ursinus* (1704) |  |  |  | *Klebsiella pneumoniae* | - |  |
| 32 | HRV(96) | HSV-1 (8) |  |  |  |  | Candida albicans(162) |  |  | HRV | *-* |
| 33 |  |  |  |  |  |  |  |  | - | - |  |
| 34 | H1N1(2888) |  |  |  |  |  |  |  |  | ADV H1N1 | *-* |
| 35 |  |  |  |  |  |  |  |  |  | - | *-* |
| 36 |  | ADV (23679) |  |  |  |  |  |  | Legionella Aspergillus fumigatus | ADV | *-* |
| 37 |  | ADV (577) |  |  |  |  |  |  | - |  |  |
| 38 |  |  |  |  | *Legionella pneumophila*(302) |  |  |  | - |  |  |
| 39 |  |  |  |  | *Streptococcus pneumoniae* (20197) *Haemophilus influenzae* (2799) |  |  |  |  |  | *Haemophilus influenzae* |
| 40 |  |  |  |  |  |  |  | *Mycoplasma pneumoniae*(140) | *Catacoccus Streptococcus hemolyticus* | - | *-* |
| 41 |  |  |  |  | *Streptococcus pneumoniae* (23623) *Haemophilus influenzae* (1442) *Streptococcus pyogenes* (111) | *Chlamydia pneumoniae*(185) |  |  |  | - | *Streptococcus pneumoniae Stenotrophomonas maltophilia Haemophilus influenzae* |
| 42 |  |  |  |  |  |  |  |  | *Catacoccus Streptococcus hemolyticus* | - | *-* |
| 43 |  |  |  |  |  |  |  |  | *Catacoccus Streptococcus hemolyticus* | - | *Pseudomonas aeruginosa Stenotrophomonas maltophilia* |
| 44 |  |  |  |  |  |  |  |  | *Coagulase-negative Staphylococcus* | - | *Pseudomonas aeruginosa Acinetobacter baumannii* |
| 45 |  |  |  |  | *Haemophilus influenzae* (53) |  |  |  |  | - | *Staphylococcus aureus* |
| 46 |  | EBV (4553) HHV-7 (67) |  |  | *Neisseria mucosa* (404) *Haemophilus influenzae*(332) *Enterococcus faecalis*(42) |  |  |  |  | - | *Legionella pneumophila Haemophilus influenzae* |
| 47 |  |  |  |  |  |  | Candida tropicalis(3723) |  |  | - | *-* |
| 48 |  |  |  |  |  |  |  |  |  | - | *-* |
| 49 |  |  |  |  |  |  |  |  |  | - | *-* |
| 50 |  | EBV (109) |  |  |  |  | Candida tropicalis(3) |  |  | - | *-* |
| 51 | HCoV-OC43 (45) |  |  |  |  |  |  |  | - | - |  |
| 52 |  |  |  |  |  |  |  |  |  | - | *-* |
| 53 |  |  |  |  | *Staphylococcus aureus*(81) |  |  |  |  | - | *-* |
| 54 | EntV D68 (8965) |  |  |  | *Streptococcus pneumoniae*(4735) |  |  |  |  | EntV | *Streptococcus pneumoniae* |
| 55 |  |  |  |  | *Staphylococcus aureus*(81) |  |  | *Mycoplasma pneumoniae*(66) |  | - | *Mycoplasma pneumoniae* |
| 56 |  |  |  |  |  |  |  |  |  | - | *-* |
| 57 |  |  |  |  |  |  |  | *Mycoplasma pneumoniae*(1108) |  | - | *-* |
| 58 |  |  |  |  | *Pseudomonas aeruginosa*(1102) |  |  | *Mycoplasma pneumoniae*(865) | *Mycoplasma pneumoniae* |  |  |
| 59 |  |  |  |  | *Pseudomonas aeruginosa*(4504),*Streptococcus pneumoniae*(3030) |  |  | *Mycoplasma saliva*(35) | *Mycoplasma pneumoniae* | - |  |
| 60 |  |  |  |  |  |  |  |  |  | - | *-* |
| 61 |  |  |  |  |  |  |  | *Mycoplasma pneumoniae*(512) |  | - | *Mycoplasma pneumoniae* |
| 62 |  |  | Mycobacterium abscessus(7) |  |  |  |  |  |  | - | *-* |
| 63 |  |  |  |  |  |  |  |  |  | - | *-* |
| 64 |  |  |  |  |  |  |  |  |  | - | *-* |
| 65 |  |  |  |  | *Streptococcus pneumoniae*(76) |  |  |  |  | - | *Streptococcus pneumoniae* |
| 66 |  |  |  |  |  |  |  |  |  | EntV | *-* |
| 67 |  |  |  |  |  |  |  |  |  |  | *-* |
| 68 |  |  |  |  |  |  | Aspergillus fumigatus(11) |  |  |  | *-* |
| 69 |  |  |  |  |  |  |  |  |  |  | *-* |
| 70 |  |  |  |  |  |  |  |  |  |  | *-* |
| 71 |  |  |  |  |  | *Chlamydia psittaci*(43) |  |  |  |  | *-* |
| 72 |  |  |  |  |  |  |  |  | - | - | *-* |
| 73 |  |  |  |  | *Pseudomonas aeruginosa*(10) |  |  | *Mycoplasma pneumoniae*(29) | - | - | *Mycoplasma pneumoniae* |
| 74 | Measles virus (30) |  |  |  | *Pseudomonas aeruginosa*(826) |  |  |  | - | - | *-* |
| 75 |  |  |  |  |  |  |  |  | - | HRV | *-* |
| 76 |  |  |  |  |  |  |  |  | - | RSV | *-* |
| 77 |  |  |  |  |  |  |  |  | - | - | *Mycoplasma pneumoniae* |
| 78 |  |  |  |  |  |  |  | *Mycoplasma pneumoniae*(302) | - | - | *Mycoplasma pneumoniae* |
| 79 |  |  |  |  |  |  |  |  | - |  | *-* |
| 80 |  |  |  |  |  |  |  |  |  | - | *-* |
| 81 |  |  |  |  |  |  |  |  | - | - | *-* |
| 82 |  |  | Mycobacterium tuberculosis complex (MTC) (1) |  |  |  |  | *Mycoplasma pneumoniae*(294) | - |  |  |
| 83 |  |  |  |  |  |  |  | *Mycoplasma pneumoniae*(157) | - |  |  |
| 84 |  |  |  | Treponema pectinosa (22) |  |  |  |  | - |  |  |
| 85 |  |  |  |  |  |  |  |  | - |  |  |
| 86 |  |  |  |  | *Pseudomonas aeruginosa*(2865) |  |  |  | *Pseudomonas maltophilia* | - | *-* |
| 87 |  |  |  |  | *Pseudomonas aeruginosa*(110) |  | Candida albicans（38） |  | Candida albicans | - | *-* |
| 88 |  |  | Mycobacterium simian complex (30) |  |  | Chlamydia psittaci(1054) | Candida albicans(61) |  | - | - | *-* |
| 89 |  |  |  |  | *Pseudomonas aeruginos*a(720) |  |  |  | Candida albicans | - | *-* |
| 90 | H3N2 (2159) |  |  |  | *Enterococcus faecalis*(156) *Pseudomonas aeruginosa*(35) |  | Candida glabrata(92) Candida tropicalis(9) |  | Candida glabrata | INFA | *-* |
| 91 |  |  |  |  | *Streptococcus pyogenes*(51) |  |  |  |  | ADV | *-* |
| 92 |  |  |  |  |  |  |  |  | MRSA | - | *Mycoplasma pneumoniae* |
| 93 | hPIV(1246) Inf B (20) |  |  |  |  |  |  |  | - | INFB | *-* |
| 94 |  |  |  |  |  |  |  |  | - | - | *-* |
| 95 |  |  |  |  |  |  |  |  | - | - | *-* |
| 96 | Measles virus (54) HRV (13) |  |  |  |  |  |  |  | - | - | *-* |
| 97 | Measles virus (2) |  |  |  |  |  |  |  | - | - | *-* |
| 98 | HRV (9) |  |  |  |  |  |  |  | - | - | *-* |
| 99 |  |  |  |  |  |  |  |  | Aspergillus | - | *-* |
| 100 |  |  |  |  |  |  |  |  | Candida krusei Candida tropicalis |  |  |
| 101 |  |  |  |  | *Staphylococcus aureus*(2461) *Pseudomonas aeruginosa*(1002) |  |  |  | *Pseudomonas aeruginosa* |  |  |
| 102 |  |  |  |  |  |  |  |  | - |  |  |
| 103 |  |  |  |  |  |  |  |  | - |  |  |
| 104 |  |  |  |  |  |  |  |  | - | hPIV | *-* |
| 105 |  |  |  |  |  |  |  | *Mycoplasma pneumoniae*(21464) |  | - | *Mycoplasma pneumoniae* |
| 106 |  |  |  |  | *Pseudomonas aeruginosa*(2071),*Streptococcus pneumoniae*(1087) |  |  |  | - | - | *Streptococcus pneumoniae Stenotrophomonas maltophilia Pseudomonas aeruginosa Haemophilus influenzae* |
| 107 |  |  |  |  | *Staphylococcus epidermidis*(37376) *Klebsiella pneumoniae(*6) |  |  | *Mycoplasma saliva*(12814) | - | - | *Klebsiella pneumoniae* |
| 108 |  |  |  |  | *Pseudomonas aeruginosa*(922) |  |  | *Mycoplasma pneumoniae*(683) | - | - | *Mycoplasma pneumoniae Pseudomonas aeruginosa* |
| 109 |  |  |  |  |  |  |  | *Mycoplasma pneumoniae*(17860) | - | - | *Mycoplasma pneumoniae* |
| 110 |  |  |  |  |  |  |  | *Mycoplasma pneumoniae*(488) | - | - | *Mycoplasma pneumoniae* |
| 111 |  |  |  |  |  |  |  | *Mycoplasma pneumoniae*(53675) | *Klebsiella oxytoca* | - | *Mycoplasma pneumoniae* |
| 112 | hMPV (9) HRV (5) |  |  |  |  |  |  | *Mycoplasma pneumoniae*(2612) | *Mycoplasma pneumoniae* | - | *Mycoplasma pneumoniae* |
| 113 |  |  |  |  |  |  |  |  | - | - | *-* |
| 114 |  |  |  |  | *Staphylococcus aureu*s(17185), |  |  |  | *Staphylococcus aureus* | - | *Staphylococcus aureus Pseudomonas aeruginosa Chlamydia pneumoniae* |
| 115 |  |  |  |  |  |  |  | *Mycoplasma pneumoniae*(1377) | *Mycoplasma pneumoniae* |  |  |
| 116 |  |  |  |  |  |  |  |  | - | - | *-* |
| 117 | HCoV-OC43(220) | HSV-1 (219) |  |  | *Streptococcus pneumoniae*(6908) *Pseudomonas aeruginosa*(170) |  |  |  | *Pseudomonas aeruginosa* | - | *-* |
| 118 |  |  |  |  |  |  |  |  | - | - | *-* |
| 119 |  |  |  |  |  |  |  |  | - | - | *-* |
| 120 |  |  |  |  |  |  |  |  | - | - | *-* |
| 121 |  | HSV-1 (6) CMV (3) |  |  |  |  |  | *Mycoplasma pneumoniae*(1012) | - | - | *Mycoplasma pneumoniae* |
| 122 |  |  |  |  |  |  |  | *Mycoplasma pneumoniae*(69994) | - | - | *Mycoplasma pneumoniae* |
| 123 | H1N1 (5105) |  |  |  |  |  |  |  | - | H1N1 | *-* |
| 124 |  |  |  |  |  |  |  |  | - |  | *-* |
| 125 |  |  |  |  | *Streptococcus pneumoniae*(16) |  |  |  | - | - | *-* |
| 126 |  |  |  |  |  |  |  |  | - | - | *-* |
| 127 |  |  |  |  |  |  | Candida albicans(3004) |  | Candida albicans | - | *-* |
| 128 |  |  |  |  |  |  |  |  | - |  |  |
| 129 |  |  |  |  | *Pseudomonas aeruginosa* (201129) |  |  |  | - |  | *Pseudomonas aeruginosa* |
| 130 |  |  |  |  |  |  |  |  | - |  | *-* |
| 131 |  |  |  |  | *Pseudomonas aeruginosa*(4) |  |  |  | - | - | *Pseudomonas aeruginosa* |
| 132 |  | EBV (1084) CMV (20) |  |  |  |  |  |  | - |  |  |
| 133 | HRV (52) |  |  |  |  |  |  |  | - |  |  |
| 134 | HRV (26) |  |  |  |  |  |  | *Mycoplasma pneumoniae*(209) | - |  |  |
| 135 | H3N2 (11) |  |  |  | *Staphylococcus aureus*(335) |  |  |  | - |  | *Staphylococcus aureus* |
| 136 |  |  |  |  |  |  |  |  | - |  |  |
| 137 |  | EBV (4600) |  |  |  |  |  |  | - |  |  |
| 138 |  | HSV-1 (586) |  |  |  |  |  |  | *Burkholderia cepacia* |  |  |
| 139 |  | HSV-1 (185) EBV(92) |  |  |  |  |  |  | - |  |  |
| 140 | hMPV (4614) |  |  |  |  |  |  |  | *Pseudomonas aeruginosa* |  | *-* |
| 141 |  | HSV-1 (12611) |  |  | *Acinetobacter baumannii(*4634) |  |  |  | *Acinetobacter baumannii* |  | *-* |
| 142 |  | EBV(77) CMV(31) |  |  |  |  |  |  |  |  | *-* |
| 143 | HRV (3) |  |  |  | *Acinetobacter baumannii*(7694) *Pseudomonas aeruginosa (*125) *Stenotrophomonas maltophilia*(29) *Klebsiella pneumoniae*(6) |  |  |  |  |  | *Klebsiella pneumoniae Acinetobacter baumannii Stenotrophomonas maltophilia* |
| 144 | H3N2 (5) | HSV1 (2478) EBV (43) |  |  | *Klebsiella pneumoniae*（70093） |  |  |  |  |  | *Klebsiella pneumoniae* |
| 145 |  |  |  |  |  |  |  |  |  |  | *-* |
| 146 |  |  |  |  | *Pseudomonas aeruginosa*(3729) *Haemophilus influenzae*(6354) |  |  |  |  |  | *Pseudomonas aeruginosa, Haemophilus influenzae* |
| 147 |  | EBV (19103) |  |  | *Klebsiella pneumoniae(*6192) *Staphylococcus ludunensis*(55701) *Streptococcus pneumoniae*(16551) *Listeria monocytogenes*(475) |  | Candida parapsilosis(417061) |  |  |  | *Klebsiella pneumoniae MRAS* |
| 148 |  |  |  |  | *Pseudomonas aeruginosa (*18481) *Haemophilus influenzae(*6699) |  | Candida albicans(32) |  |  |  | *Pseudomonas aeruginosa, Haemophilus influenzae* |
| 149 | hMPV (140357) |  |  |  | *Streptococcus pneumoniae*(36616) |  | Candida albicans（66） |  |  |  | *Streptococcus pneumoniae, Haemophilus influenzae* |
| 150 |  |  |  |  |  |  |  |  |  |  | *-* |
| 151 |  |  |  |  | *Haemophilus influenzae(*11020) |  | Cryptococcus neoformans(7) |  |  |  | *-* |

TableS7 Pathogen detection in 11 samples collected within six months

| **Case ID** | **LAMP** | **DNA mNGS (reads)** | **RNA mNGS（reads）** | **qPCR** |
| --- | --- | --- | --- | --- |
| 144 | *K. pneumoniae* | *K. pneumoniae*（70093） HSV1（2478 EBV（43） | H3N2（5） | *K. pneumoniae*-Ct-21.86 |
| 145 | / | / | / | / |
| 149 | *S. pneumoniae* *H. influenzae C. albicans*-NOT DETECTED | *S. pneumoniae*（36616） *C. albicans*（66） *H. influenzae*（58） | hMPV（140357） | *K. pneumoniae*-Ct-28.34；  *C. albicans*-Ct-33.18；  *H. influenzae*-NOT DETECTED； |
| 150 | / | / | / |  |
| 143 | *K. pneumoniae A. baumannii S. maltophilia* | *A. baumannii*（7694） *P. aeruginosa*（125） *S. maltophilia*（29） *K. pneumoniae*（6） | HRVs C11 CL-170085（3） | *S. maltophilia*-Ct-21.06； *K. pneumoniae*-NOT DETECTED； |
| 148 | *P. aeruginosa H. influenzae* | *P. aeruginosa*（18481） *H. influenzae*（6699） *C. albicans*（32） | / | *H. influenzae*-Ct-24.78； *C. albicans*-Ct-34.02 |
| 151 | *H. influenzae*-NOT DETECTED | *H. influenzae*（11020） *C. neoformans*（7） | / | *H. influenzae*-Ct-28.74； |
| 146 | *P. aeruginosa H. influenzae* | *P. aeruginosa*（3729） *H. influenzae*（6354） | / | *H. influenzae*-Ct-24.82； |
| 142 | / | EBV(77) CMV（31） | / | / |
| 147 | *S. pneumoniae*-NOT DETECTED *K. pneumoniae* | EBV（19103） *K. pneumoniae*（6192） *S. lugdunensis*（55701） *S. pneumoniae*（16551） *L. monocytogenes*（475） *C. glabrata*（417061） | / | *S. pneumoniae*-Ct-23.52； *K. pneumoniae*-Ct-27.29； |
| 39 | *S. pneumoniae*-NOT DETECTED *H. influenzae* | *S. pneumoniae*（20197） *H. influenzae*（2799） | / | *S. pneumoniae*-Ct-28.62； *H. influenzae*-Ct-31.03； |

# Supplement 4 Supplementary Figures


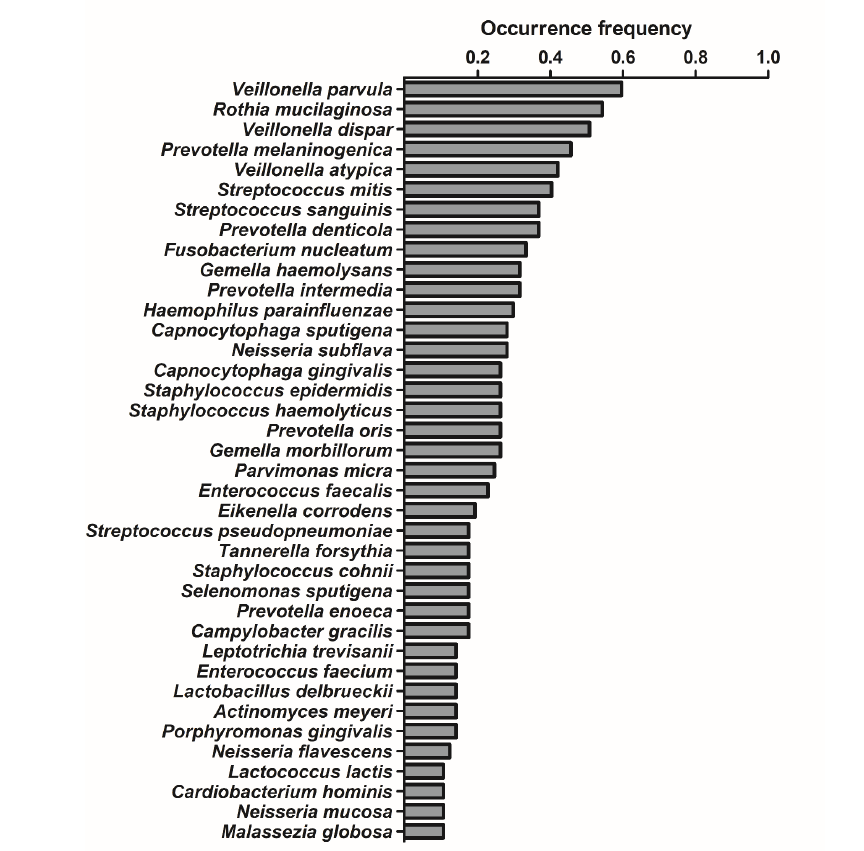


Supplementary Figure 1. Spectrum of background flora. Occurrence frequency of species detected among respiratory specimens from patients with non-infectious disease.


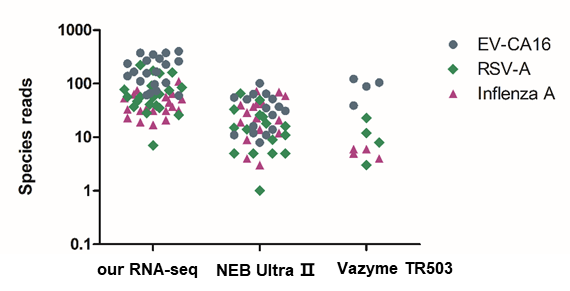


Supplementary Figure 2. mNGS assay optimization on RNA library preparation procedures. Comparison of our RNA library preparation method with two commercial Kits, NEBNext Ultra II RNA Library Prep Kit for Illumina and Vazyme TR503 TruePrep® RNA Library Prep Kit for Illumina. BALF samples spiked with Enterovirus coxsackievirus A16 (EV-CA16), Respiratory Syncytial Virus A (RSV-A), Influenza A virus were used for RNA library preparation with three different methods. After sequencing with 5M for each library, species reads were calculated respectively. Values represent different BALF samples (n=20 for our RNA-seq method, n=16 for NEB kit and n=4 for Vazyme kit).


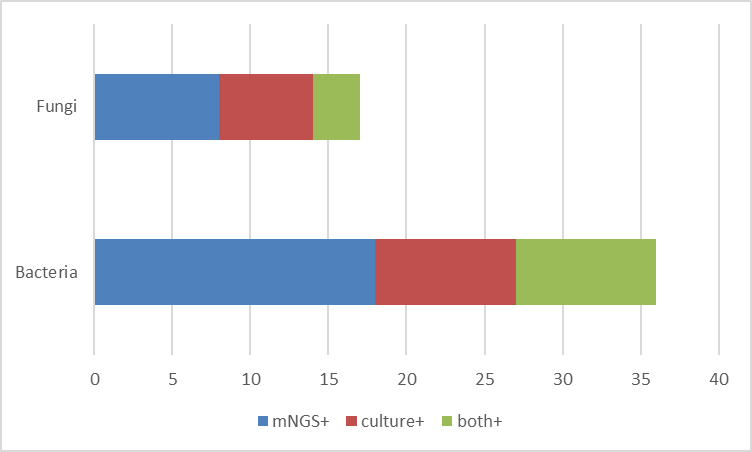


Supplementary Figure 3. The overlap of positivity between mNGS and culture for fungi and bacteria. Both+, results of mNGS and culture were both positive; mNGS+, only the mNGS result was positive, culture was not; culture+, only the culture result was positive, mNGS was not.
